# Advances in the Genus *Ulva* Research: From Structural Diversity to Applied Utility

**DOI:** 10.3390/plants14193052

**Published:** 2025-10-02

**Authors:** Thanh Thuy Duong, Hang Thi Thuy Nguyen, Hoai Thi Nguyen, Quoc Trung Nguyen, Bach Duc Nguyen, Nguyen Nguyen Chuong, Ha Duc Chu, Lam-Son Phan Tran

**Affiliations:** 1 Faculty of Agronomy, University of Agriculture and Forestry, Hue University, Hue City 530000, Vietnam; duongthanhthuy@hueuni.edu.vn (T.T.D.); nthoai.huaf@hueuni.edu.vn (H.T.N.); 2 Faculty of Fisheries, University of Agriculture and Forestry, Hue University, Hue City 530000, Vietnam; ntthang.dhnl@hueuni.edu.vn; 3 Faculty of Biotechnology, Vietnam National University of Agriculture, Hanoi City 100000, Vietnam; nqtrung@vnua.edu.vn (Q.T.N.); ndbach@vnua.edu.vn (B.D.N.); 4 Department of Plant and Soil Science, Institute of Genomics for Crop Abiotic Stress Tolerance, Texas Tech University, Lubbock, TX 79409, USA; chuonguy@ttu.edu; 5 Faculty of Agricultural Technology, University of Engineering and Technology, Vietnam National University Hanoi, Hanoi City 100000, Vietnam

**Keywords:** economic application, molecular taxonomy, morphology, sea lettuce, algal bloom

## Abstract

The green macroalgae *Ulva* Linnaeus, 1753, also known as sea lettuce, is one of the most ecologically and economically significant algal genera. Its representatives occur in marine, brackish, and freshwater environments worldwide and show high adaptability, rapid growth, and marked biochemical diversity. These traits support their ecological roles in nutrient cycling, primary productivity, and habitat provision, and they also explain their growing relevance to the blue bioeconomy. This review summarizes current knowledge of *Ulva* biodiversity, taxonomy, and physiology, and evaluates applications in food, feed, bioremediation, biofuel, pharmaceuticals, and biomaterials. Particular attention is given to molecular approaches that resolve taxonomic difficulties and to biochemical profiles that determine nutritional value and industrial potential. This review also considers risks and limitations. *Ulva* species can act as hyperaccumulators of heavy metals, microplastics, and organic pollutants, which creates safety concerns for food and feed uses and highlights the necessity of strict monitoring and quality control. Technical and economic barriers restrict large-scale use in energy and material production. By presenting both opportunities and constraints, this review stresses the dual role of *Ulva* as a promising bioresource and a potential ecological risk. Future research must integrate molecular genetics, physiology, and applied studies to support sustainable utilization and ensure safe contributions of *Ulva* to biodiversity assessment, environmental management, and bioeconomic development.

## 1. Introduction

The genus *Ulva* Linnaeus, 1753, comprises green macroalgae that are characterized by thin, sheet-like or tubular thalli [1]. Species in this genus exhibit diverse morphological forms and typically appear vibrant green due to the presence of abundant chlorophyll within their cells. They are primarily found in marine environments, especially in intertidal zones and shallow subtidal waters, as well as in brackish and freshwater habitats. Additionally, they hold considerable economic importance and have traditionally served as food in multiple cultures. Recently, they have gained attention for industrial uses in agriculture, pharmaceuticals, biofuel production, and wastewater treatment [2,3]. Members of the genus *Ulva* are widely distributed globally, occurring along temperate, tropical, and subtropical coasts. They particularly thrive in nutrient-rich waters, which are often influenced by anthropogenic activities or natural upwelling phenomena. Due to their extensive global distribution and ecological adaptability, *Ulva* species represent critical ecological indicators or biomonitors and possess substantial potential for sustainable economic applications [4].

Of particular relevance to this review is that the taxonomy of *Ulva* species primarily relied on morphological characteristics, which often resulted in misidentifications due to high phenotypic plasticity [5]. However, the advent and widespread adoption of molecular genetic techniques have significantly enhanced the accuracy of species identification and provided greater clarity to phylogenetic relationships within this genus [6]. Precise taxonomic delineation of *Ulva* species is crucial not only for understanding their ecological roles but also for harnessing their full economic potential [6,7]. Previous reports demonstrated that *Ulva* species are key contributors to coastal marine environments, as they significantly influence primary production, nutrient dynamics, and habitat complexity [2,7]. Notably, excessive proliferation of *Ulva*, referred to as green tides, can cause habitat degradation and oxygen depletion [8]. These impacts demonstrate the need for comprehensive ecological studies to support effective management and mitigation strategies [9]. Although not all species are cosmopolitan and some are endemics, the wide distribution of *Ulva* nevertheless offers considerable opportunities for sustainable resource utilization [3]. Although interest in *Ulva* species has grown in recent years, existing reviews have largely addressed individual aspects, such as taxonomy, ecology, or economic use in isolation [3,4,10,11]. An updated synthesis that integrates these dimensions, particularly in light of recent molecular insights, growth dynamics, and applied innovations, remains limited.

The purpose of this review is to deliver a thorough and critical synthesis of recent progress in the taxonomic characterization, ecological understanding, and economic exploitation of *Ulva* species. By bringing together advances in morphological and molecular systematics, insights into ecosystem functions and growth dynamics, and emerging industrial applications, from food and feed to bioremediation and bioenergy, we seek to highlight persistent knowledge gaps and establish clear priorities for future research aimed at sustainable management and utilization of this globally distributed macroalgal resource.

## 2. Morphological Variability and Environmental Plasticity in *Ulva* Species

### 2.1. Morphological Diversity of Ulva Species

**Figure 1:**
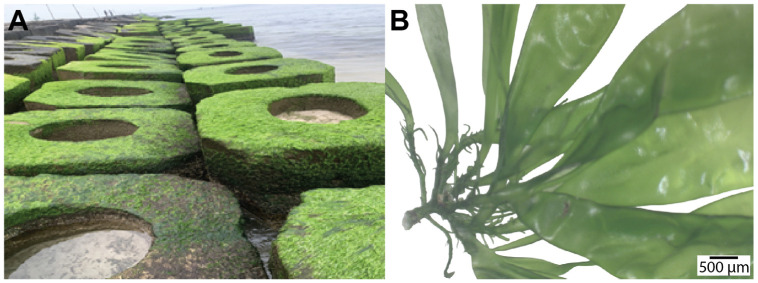
Example of *Ulva* artificial habitat in Vietnam. (**A**) Field photograph showing *Ulva* species attached to shoreline protection structures made of concrete blocks along the coast in Vietnam, specifically at the sampling site located at 16°34′23.9″ N, 107°37′05.9″ E. (**B**) The thalli display a combined tubular-leaf-like morphology typical of *Ulva* species. Light microscopy reveals thallus structure of a specimen.

Morphological characteristics, particularly thallus structure, represent fundamental criteria historically used to differentiate and classify species within the genus *Ulva* [12,13]. Traditionally, these species were divided into two primary morphological groups based on their distinct thallus structures, including sheet-like and tubular forms (Figure 1) [5,14]. Species possessing flattened, foliaceous thalli have been classically assigned to the genus *Ulva*, while those exhibiting hollow, tubular thalli were formerly categorized under the genus *Enteromorpha* Link, 1820 [15]. However, molecular evidence from earlier studies refuted the distinct separation based on morphology. This led to the reclassification of *Enteromorpha* species under the genus *Ulva* [16]. Despite this taxonomic revision, morphological distinctions are still widely described in regional algological keys. While these features can assist with preliminary field identification, accurate classification increasingly relies on molecular approaches to overcome the limitations of phenotypic plasticity. Thus, this section specifically examines and compares the morphological characteristics of the sheet-like and tubular forms. It emphasized the complexity, plasticity, and ecological implications associated with these distinct thallus architectures. The sheet-like thallus represents one of the most distinctive structural forms within the genus Ulva. Species presenting this morphology are characterized by expansive, membranous thalli resembling broad, flat blades [8,13]. The thallus typically comprises two cellular layers, each containing distinct chloroplasts and pyrenoids, thus imparting a vivid green coloration [17,18,19]. Depending on environmental variables such as nutrient concentration, salinity levels, temperature fluctuations, water turbulence, and stage of growth, these sheet-like thalli exhibit substantial variation in size, extending from a few centimeters to greater than one meter in length [20]. Among these, *U. lactuca* Linnaeus, 1753 is prominently studied due to its broad geographic distribution across temperate and subtropical coastal habitats [8,9]. The thalli of *U. lactuca* are typically thin, smooth, and translucent, with irregular shapes and flexible structures. This variability considers a high degree of morphological plasticity influenced by environmental conditions [19]. Another representative species, *U. rigida* C. Agardh, displays comparatively strong and thicker blades [21], typically characterized by wrinkled edges and commonly inhabiting rocky substrates exposed to tidal actions [21,22]. The sheet-like thallus architecture provides various ecological adaptations beneficial for the organism’s survival. The relatively large surface area in proportion to biomass allows for the efficient uptake of essential nutrients and the effective use of light [23], which enhances photosynthetic performance, especially in shallow and nutrient-rich environments [3,8]. Conversely, this morphological feature increases susceptibility to mechanical fragmentation caused by wave disturbances and herbivore grazing, although such fragmentation facilitates vegetative reproduction and extensive geographic dispersal. From an ecological standpoint, sheet-like *Ulva* species, depending on their stage of growth, can form dense accumulations, particularly in nutrient-enriched environments. These large-scale growth events can cause significant ecological disruption and economic loss. The accumulation of decomposing algal biomass often leads to oxygen depletion, which adversely affects benthic communities and reduces biodiversity.

The tubular form within the *Ulva* genus is characterized by a distinct thallus structure, wherein the algae grow as hollow, cylindrical, tube-like shapes rather than the broad, flat blades observed in sheet-like species [24] (Figure 1). This morphology often results in long, tubular filaments that can vary in diameter but typically maintain a uniform width along their length. The thallus of tubular *Ulva* species is typically composed of a single layer of cells that encircle a central lumen, a characteristic feature that differentiates it from the sheet-like forms [25]. The tubular shape provides several advantages, particularly in areas with strong water movement, as it minimizes the resistance against currents and facilitates the efficient transport of water and nutrients through the lumen [25]. One example of a tubular *Ulva* species is *U. flexuosa* Wulfen, which is typically found in coastal waters with high hydrodynamic activity [26,27]. This *Ulva* species possesses delicate, thread-like structures that are highly flexible [27]. This flexibility enables the species to withstand the mechanical stress imposed by waves and tides. Another example is *U. prolifera* O.F. Müller, a species with tubular thalli [14,28]. This species often occurs in sheltered intertidal zones, where its rapid growth and extensive tubular structures may provide advantages in nutrient absorption and space utilization [29,30,31]. The tubular morphology offers several ecological and physiological benefits. The cylindrical shape increases the surface-to-volume ratio and thereby improves nutrient uptake and gas exchange. In addition, the hollow thallus structure increases buoyancy, which allows the algae to float or remain suspended in the water column and enhances both light capture and dispersal potential [29,30,31]. Additionally, tubular *Ulva* species typically exhibit rapid growth rates and high reproductive output [23,32].

These characteristics contribute to the expansive colonization potential of tubular Ulva species, particularly under nutrient-enriched conditions. However, this morphology has important ecological implications. The tubular thalli, while reducing drag in flowing water, are highly susceptible to desiccation during low tides or periods of reduced water coverage [23]. The hollow structure also provides microhabitats for diverse marine invertebrates such as amphipods (*Gammarus locusta*), polychaetes (*Hediste diversicolor*), and gastropods (*Littorina littorea*), which shelter within the thalli and influence algal population dynamics through grazing and detritus processing [33]. Tubular species, including *U. intestinalis* Linnaeus and *U. prolifera*, often form dense accumulations. These mats create habitat for associated fauna but may also drive ecological degradation once biomass decomposes. The decomposition process depletes oxygen, lowers redox potential, and promotes the release of nutrients and toxic gases such as hydrogen sulfide (H_2_S) and ammonia (NH_3_). These changes alter nitrogen cycling and reduce benthic biodiversity [34].

### 2.2. Effects of Salinity, Temperature, and Nutrient Availability on the Morphology of Ulva Species

The taxonomy of the genus *Ulva* presents challenges, as many species exhibit high phenotypic plasticity and lack consistently reliable external morphological traits. Nevertheless, some species can be readily recognized by macroscopic features, while others are distinguishable through microscopic criteria such as cell arrangement within the thallus or the number of pyrenoids per cell. This plasticity allows *Ulva* to adapt to changes in adverse environmental conditions by altering its morphology [24]. Environmental factors, such as salinity, temperature, light, and nutrient availability, have a strong influence on the morphology of *Ulva* species [30,35,36,37]. For instance, when nutrients are abundant, *U. prolifera* may develop larger, denser thalli, while in nutrient-poor environments, it may produce smaller and more delicate structures [14,30]. Fluctuations in salinity can significantly influence the composition of the cell wall in *Ulva* species [35,36]. These changes in cell wall composition subsequently result in variations in texture and flexibility. These morphological changes are essential for the algae’s survival in diverse conditions but complicate the process of species identification and classification. Thus, the remarkable adaptability of *Ulva* necessitates the use of molecular tools, in addition to traditional morphological methods, to accurately classify and differentiate species [17,38].

Salinity plays a crucial role in shaping the morphology of *Ulva* species [39]. It influences various aspects of their growth and structural development. *Ulva* species exhibit significant morphological plasticity in response to changes in salinity, which affects the physiological processes of the algae, including cell division, osmoregulation, and turgor pressure [39,40,41]. In general, elevated salinity levels above 35–40 Practical Salinity Unit (PSU) reduce the growth rate of *Ulva* species and lead to smaller, thicker thalli. For example, in *U. prolifera*, salinity treatments above 40 PSU caused a significant decline in relative growth rate and increased thallus thickness from approximately 55–65 µm at 30 PSU to 80–95 µm at 45 PSU [34]. This adaptation helps *Ulva* maintain cellular turgor pressure and prevent excessive water loss through osmosis [42]. On the other hand, at reduced salinity levels below 30 PSU, many *Ulva* species exhibit higher growth rates and develop thinner, more delicate thalli, as osmotic stress is diminished and water and nutrient uptake become more efficient. For instance, *U. lactuca* maintains rapid growth at 15–25 PSU with thallus thickness around 50–60 µm, but biomass accumulation drops substantially in near-freshwater conditions of 0.5–1 PSU [43]. It reported changes in the morphological plasticity of *U. prolifera* under different laboratory environmental conditions and demonstrated conclusively that *U. prolifera* can acclimatize to ecological changes via morphology-driven physiological and biochemical adjustments [35]. It has been observed remarkable morphological acclimation of *U. prolifera* to varying salinity levels (10, 20, and 30‰) [35]. Specifically, when cultured at lower salinities, *U. prolifera* developed more numerous and shorter branches. This increased branching may result in a higher net photosynthetic rate due to enhanced synthesis of photosynthetic pigments under low salinity conditions. Increased thallus density also helped protect the organism from osmotic stress caused by low salinity [35]. The maximum growth rate of *U. pertusa* was observed at 20‰ salinity, and the minimum at 40‰ under experimental conditions across six salinity levels (5, 15, 20, 25, 34, and 40‰) [41]. This suggests that *U. pertusa* has a broader salinity tolerance than strictly marine algae [41]. However, the distribution of *U. compressa* L. appears wider than previously reported; recent molecular and morphological studies confirm its presence in Baltic Sea waters with salinities as low as ~5–15 PSU, particularly in drifting or sheet-like morphotypes rather than strictly tubular forms [44]. Also, salinity significantly influenced chlorophyll *a* content, effective quantum yield, and nutrient uptake in these *Ulva* species [41,42,45]. These morphological adaptations are critical for enhancing the algae’s ability to cope with osmotic stress and optimize resource acquisition under different environmental conditions [39,40,41]. In addition to marine and brackish representatives, several *Ulva* species inhabit freshwater ecosystems, a group often overlooked in phycological studies [46]. These taxa demonstrate the ability of *Ulva* to persist under low salinity conditions (<0.5 PSU). They broaden the ecological amplitude of the genus and emphasize the need to integrate freshwater representatives into biodiversity and biogeography assessments.

Temperature has a profound effect on the morphology of *Ulva* species. It influences their growth, development, and structural characteristics [35]. As poikilothermic organisms, *Ulva* species are highly sensitive to temperature fluctuations, which can trigger a range of morphological changes [12,35]. At higher temperatures, *Ulva* species often exhibit increased growth rates, leading to the production of larger thalli. However, prolonged exposure to elevated temperatures can also result in thinner and more fragile thalli, as the algae may experience thermal stress and reduced cell wall strength. On the other hand, low temperatures typically slow down growth rates. This results in smaller, more compact thalli with denser cell walls. These temperature-induced morphological changes are an adaptive response to the stress imposed by variations in thermal conditions. They allow the algae to optimize resource allocation and energy use [47,48]. Temperature affected the growth and photosynthetic responses of *U. prolifera* under laboratory conditions [35,47]. The photosynthetic rate was significantly higher at moderate temperatures (14–27 °C), while both low (<14 °C) and high (40 °C) temperatures had negative effects on photosynthesis [35]. It found that *U. lactuca* exhibited accelerated growth and larger thalli at higher temperatures, particularly in nutrient-rich environments [49]. However, when exposed to temperatures exceeding 30 °C, the species showed signs of stress, including reduced thallus thickness and cell integrity [50]. Additionally, *U. rigida* has been shown to modify its thallus structure when exposed to varying temperature conditions, with higher temperatures resulting in longer and more elongated thalli, whereas lower temperatures caused the algae to produce shorter, more vigorous blades [51]. These morphological responses demonstrate the importance of temperature in shaping the physical characteristics and overall fitness of *Ulva* species, especially in coastal environments where temperature fluctuations are common due to seasonal changes and climate variability [12,35,52].

Nutrient availability significantly influences the morphology of *Ulva* species by affecting aspects such as thallus size, thickness, and branching patterns [14]. In environments with high nutrient concentrations, *Ulva* species often develop larger and stronger thalli [53]. This morphological adaptation enhances the algae’s capacity for nutrient uptake and photosynthesis, thereby supporting rapid growth and proliferation. Conversely, in nutrient-poor conditions, *Ulva* thalli tend to be smaller and thinner. This pattern reflects a strategic allocation of limited resources to maintain essential physiological functions [54]. For instance, research on *U. prolifera* demonstrated that elevated levels of dissolved inorganic nitrogen and phosphorus led to increased growth rates and biomass accumulation, accompanied by noticeable changes in thallus morphology [55]. Specifically, the algae developed broader and thicker blades under nutrient-rich conditions, which facilitated more efficient light capture and nutrient assimilation [55]. Similarly, studies on *U. lactuca* have shown that nutrient enrichment results in the formation of denser and more expansive thalli, while nutrient limitation leads to reduced thallus expansion and a more compact structure [20]. These morphological adaptations are not merely structural but are closely linked to the algae’s ecological strategies. In nutrient-rich environments, the expansive growth of *Ulva* can lead to the formation of dense algal accumulations, which can have significant ecological and economic impacts [47,55,56,57]. Understanding the relationship between nutrient availability and *Ulva* morphology is therefore crucial for predicting large-scale growth dynamics and managing coastal ecosystems affected by eutrophication.

### 2.3. Reproductive Strategies and Environmental Adaptability of Ulva Species

**Figure 2:**
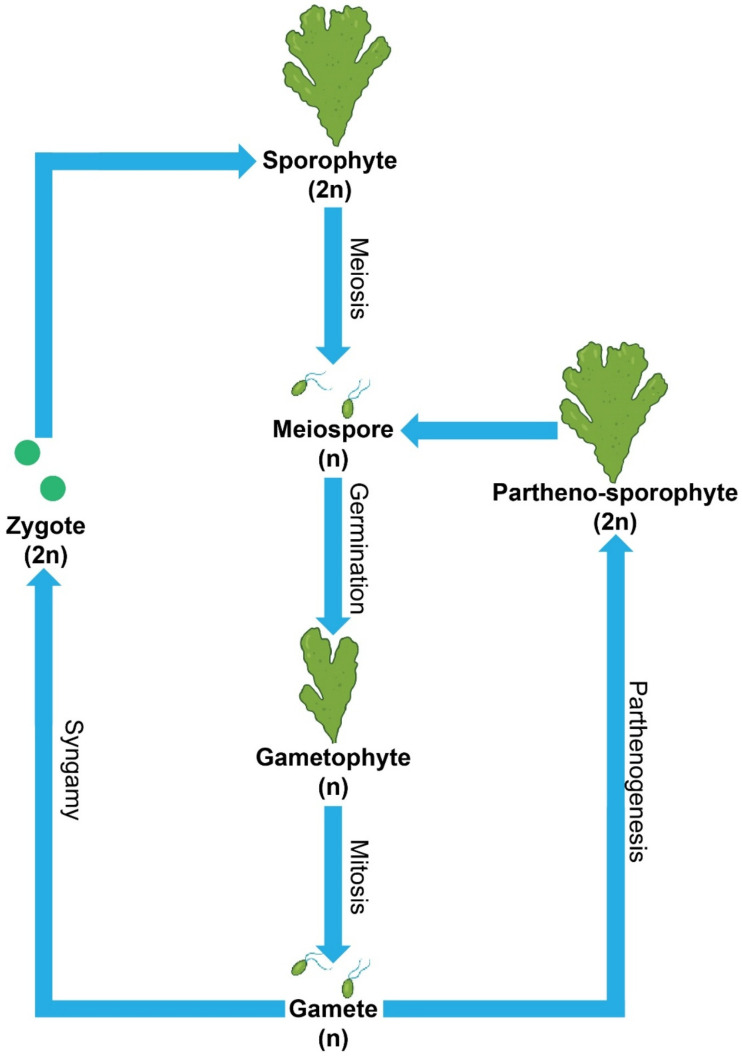
General reproduction strategies of *Ulva* species.

*Ulva* exhibits both sexual and asexual reproduction. The sexual cycle follows an isomorphic alternation of generations (Figure 2). Haploid gametophytes (1n) release biflagellate gametes that fuse to form diploid zygotes (2n). These zygotes develop into diploid sporophytes (2n), which undergo meiosis to release meiospores (1n). The meiospores then grow into gametophytes (1n), completing the cycle [29]. This alternation maintains genetic diversity and allows *Ulva* populations to adapt to changing environmental conditions [35]. Asexual reproduction occurs through thallus fragmentation or spore production. Detached thallus fragments regenerate into new individuals, and zoospores (1n) disperse widely, which accelerates colonization of coastal habitats. In addition, *Ulva* reproduces by parthenogenesis, where gametes (1n) develop directly into new thalli. In *U. prolifera* from the Yellow Sea, some strains produced parthenosporophytes (2n) that alternated with gametophytes (1n), while other strains produced successive gametophyte generations (1n) before reverting to the gametophyte-parthenosporophyte cycle [29,58,59]. This range of reproductive modes supports the persistence and rapid spread of *Ulva*, particularly during bloom formation [28,45,47,48].

Environmental factors strongly influence phase transitions between gametophyte (1n) and sporophyte (2n) stages in *Ulva* [60]. Temperature, salinity, and nutrient availability determine the predominance of each phase [41,48,55]. In *U. prolifera*, high temperature and nutrient enrichment promote the sporophyte stage, which produces more zoospores and drives population expansion [47]. Stress conditions favor the gametophyte stage and stimulate sexual reproduction that increases genetic diversity. The isomorphic alternation of generations allows both phases to occupy the same niches and maintain growth in unstable habitats. This trait supports rapid colonization of eutrophic coasts and underlies the development of green tides [47,55,61].

**Figure 3:**
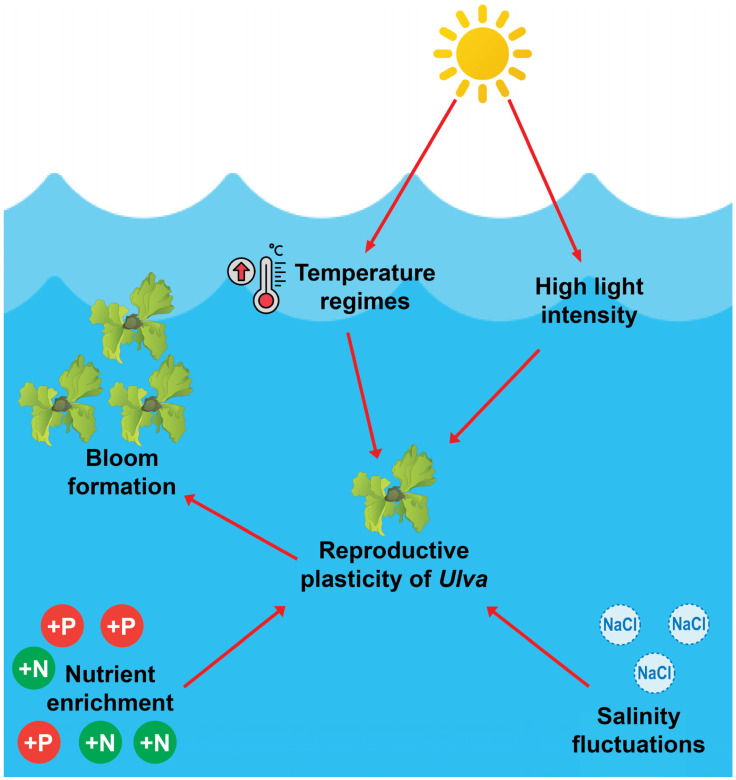
Dynamics of development and growth of marine species of *Ulva*.

Large accumulations of *Ulva* occur as large accumulations of biomass in nutrient-rich waters and result mainly from eutrophication caused by agriculture, wastewater, and aquaculture (Figure 3). *Ulva prolifera*, *U. linza*, and *U. compressa* often dominate these events because of fast growth, efficient nutrient uptake, and reproduction through both sexual and asexual modes [24,28,30,47]. Fragmentation and zoospore release support rapid multiplication and wide colonization [56,62]. In the Yellow Sea, *U. prolifera* uses these strategies to sustain recurrent large-scale accumulations of biomass [30]. The combination of phenotypic plasticity and an isomorphic life cycle strengthens survival across varied conditions [54,55]. Blooms reduce oxygen levels through algal decay, disrupt benthic communities, cause fish kills, and may spread pathogenic bacteria. They also damage tourism and fisheries [58,63]. Effective management depends on reducing nutrient inputs with better farming practices, improved wastewater treatment, and aquaculture regulation [36,47,48,55].

## 3. Economic Application of *Ulva* Species

The economic potential of *Ulva* is closely linked to its biochemical composition, which provides the foundation for its diverse applications. *Ulva* species exhibit a complex biochemical profile consisting of carbohydrates, proteins, lipids, pigments, vitamins, minerals, and bioactive compounds that together underpin both nutritional and industrial value [64]. Carbohydrates constitute the largest fraction of the dry weight, typically ranging from 47% to 67% [4]. These include starch (~4%), cellulose (9–10%), hemicellulose (14–32%), and the sulfated polysaccharide ulvan (13–39%). Ulvan has attracted considerable attention due to its diverse biological activities, including antioxidant, anticoagulant, immunomodulatory, and antitumor properties, which make it a promising candidate for pharmaceutical and biotechnological applications [4,10,65,66,67]. Proteins typically represent 10–30% of the dry weight and are characterized by a balanced amino acid profile, containing essential amino acids such as leucine, lysine, and valine [64]. Although the lipid fraction is relatively low (0.2–4%), *Ulva* species are notable for their enrichment in polyunsaturated fatty acids, particularly omega-3 and omega-6 fatty acids, which contribute to both human nutrition and animal feed applications [64,68,69]. *Ulva* is also a valuable source of minerals and trace elements. It contains appreciable amounts of sodium (351–364 mg/100 g), potassium (209–467 mg/100 g), and calcium (180–1828 mg/100 g). Trace minerals such as iron (14–34 mg/100 g), zinc, copper (both ~1.8 mg/100 g), selenium (1.6 mg/100 g), and manganese (4.8 mg/100 g) further enhance its nutritional profile. In addition, *Ulva* is rich in vitamins, including A (0.5 IU/100 mg), several B-complex vitamins (B1, B2, B3, and B12), vitamin C (2–90 ppm), and vitamin E (25.8 mg/kg dry weight) [21,69]. Additionally, chlorophylls a and b, together with carotenoids such as lutein and β-carotene, contribute to antioxidant capacity and human health benefits [28,64]. The combination of these nutritional and functional compounds not only contributes to ecosystem productivity but also reinforces *Ulva*’s role as a sustainable bioresource.

**Figure 4:**
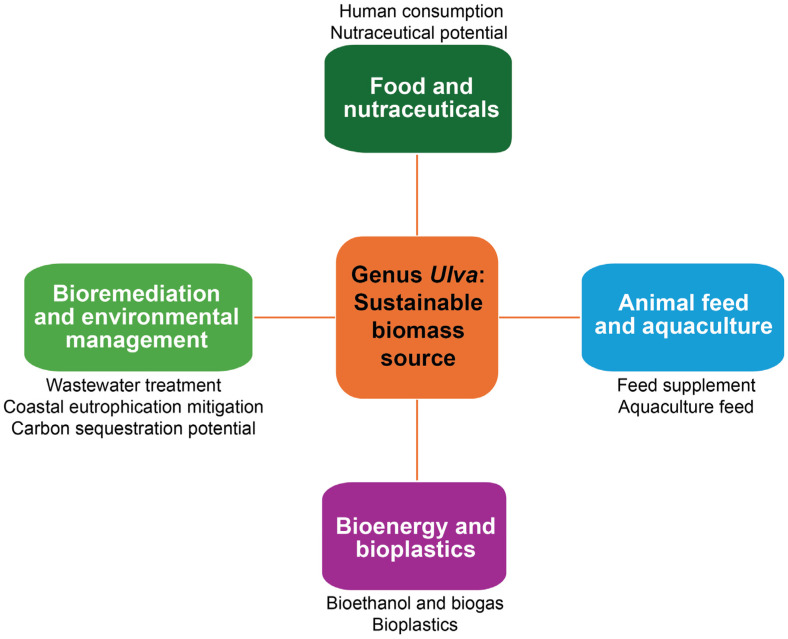
Overview of the key economic applications of *Ulva* biomass.

*Ulva* species have garnered significant attention in aquaculture and bioengineering due to their rapid growth rates, environmental adaptability, and potential for biomass production (Table 1, Figure 4). Their cultivation in controlled systems offers opportunities for optimizing biomass yield and exploring commercial applications, such as biofuel production and bioremediation [3,50]. In aquaculture settings, *Ulva* species are cultivated using various systems, including land-based tanks, raceways, and offshore platforms. These systems allow for the control of environmental parameters to enhance growth and biomass accumulation. For instance, *U. lactuca* has been successfully cultivated in integrated multitrophic aquaculture systems, where it utilizes nutrients from fish effluents, thereby improving water quality and producing valuable biomass [8,50]. Similarly, *U. fenestrata* has demonstrated the ability to complete its life cycle under laboratory conditions [70]. This finding indicates its potential for sustainable aquaculture practices. Optimizing biomass production in *Ulva* cultivation involves manipulating factors, such as nutrient availability, light intensity, and stocking density. Studies have shown that controlled reproduction and growth conditions can lead to increased biomass yields. For example, cultivating *U. lactuca* with liquid manure as a nutrient source resulted in biomass suitable for bioenergy production and as a protein-rich feed supplement [8,71]. Additionally, understanding the dynamics of sporulation and implementing strategies to manage it can prevent sudden biomass losses and ensure consistent production. The commercial exploitation of *Ulva* biomass extends to applications in biofuel production and bioremediation. *Ulva* species have been identified as suitable candidates for biofuel due to their high carbohydrate content and rapid growth rates [72]. Moreover, their ability to absorb nutrients and heavy metals from wastewater makes them effective agents for bioremediation [2,70]. Particularly, *U. prolifera* has been utilized in laboratory-scale photobioreactors to treat coking effluent. This application demonstrated significant removal efficiencies for nitrogen, phosphorus, and heavy metals [28,35,47,48]. Similarly, *U. lactuca* has shown potential in mitigating eutrophication by bioaccumulating heavy metals and nutrients in polluted coastal areas [9].

*Ulva lactuca* has been traditionally consumed in East Asian cuisines, particularly in Japan, Korea, and China. In Japan, it is referred to as “aosa” and is commonly used in soups, salads, and as a garnish for various dishes. In Korea, known as “parae,” it is incorporated into soups and side dishes. In China, sea lettuce is utilized in soups and stir-fries, valued for its delicate flavor and nutritional benefits [8]. *U. lactuca* is rich in proteins, dietary fibers, and essential minerals. Studies have shown that it contains approximately 13.6% protein, 28.4% dietary fiber, and 58.1% carbohydrates. It is also a good source of vitamins A, B1, and B2, and minerals such as calcium, magnesium, and iron [69,73]. Beyond its nutritional value, *U. lactuca* possesses bioactive compounds with potential health benefits [69]. These include antioxidants, anti-inflammatory, and antimicrobial properties, attributed to compounds like polyphenols, flavonoids, and carotenoids. Such properties suggest its potential use in nutraceuticals and functional foods aimed at promoting health and preventing diseases [8,69].

Next, *Ulva* species, notably *U. lactuca* and *U. ohnoi* Hiraoka & Shimada, have garnered attention in animal nutrition and aquaculture due to their rich nutrient profiles and environmental benefits. Incorporating *Ulva* biomass into livestock and poultry diets has demonstrated improvements in growth performance and health. For instance, dietary inclusion of *U. lactuca* in poultry feed has been associated with enhanced antioxidant status, reduced oxidative stress, and improved immune responses [74]. This is attributed to the presence of bioactive compounds, such as phenolics and flavonoids, in *Ulva* species [68,69,73]. Additionally, the antimicrobial properties of *Ulva* can contribute to better gut health. These properties inhibit pathogenic bacteria, like *Salmonella*, *Staphylococcus aureus*, and *Escherichia coli* [72,75]. In aquaculture, *Ulva* serves as a valuable feed component for various species [71,74]. Studies have shown that incorporating *U. lactuca* into fish diets can replace up to 100% of fishmeal without adverse effects on growth performance [50]. Specifically, in juvenile gilthead seabream (*Sparus aurata*), diets supplemented with *Ulva* from integrated multi-trophic aquaculture systems have maintained growth rates comparable to traditional feeds [76]. Also, *U. ohnoi* has been assessed as a feed ingredient. Recent study has shown that its inclusion does not negatively affect food acceptance or growth performance in fish species [76].

One of the major economic applications of *Ulva* species is a promising candidate for sustainable bioenergy and bioplastic production due to their high carbohydrate content and unique polysaccharides [77,78]. The high carbohydrate content of *U. lactuca*, approximately 55–60% [8,69], makes it an excellent feedstock for bioethanol production [79]. The process involves pretreatment, enzymatic hydrolysis, and fermentation to convert the biomass into ethanol. For instance, it demonstrated that oil-extracted residual biomass of *U. lactuca* could be effectively used for bioethanol production. The process optimizes yield through separate hydrolysis and fermentation steps [68,79]. Additionally, the low lignin content in *Ulva* species facilitates the easier breakdown of biomass. This characteristic enhances the efficiency of biofuel production [78]. Furthermore, ulvan, a sulfated polysaccharide extracted from *Ulva* species, has garnered attention for its potential in bioplastic production [65]. Its unique chemical structure allows for the formation of biodegradable films suitable for packaging applications. Studies have demonstrated that ulvan-based films possess favorable properties, including flexibility and biodegradability. These characteristics position them as viable alternatives to conventional plastics. In addition, ulvan has been converted into edible films, which support the development of sustainable food packaging solutions [66].

*Ulva* species, notably *U. lactuca* and *U. prolifera*, have demonstrated significant potential in bioremediation and environmental management due to their rapid growth rates, high nutrient uptake capacities, and adaptability to various environmental conditions. Particularly, *Ulva* species are effective in removing excess nutrients and heavy metals from wastewater. For instance, *U. prolifera* has been tested under laboratory conditions for the treatment of coking effluent, with removal efficiencies of up to 26.1% for total nitrogen and 68.5% for total phosphorus within 24 h [80]. Additionally, it showed high removal efficiencies for certain heavy metals, chemical oxygen demand, and biological oxygen demand [80]. Similarly, experiments conducted under controlled laboratory conditions demonstrated that nitrogen removal efficiencies of *U. lactuca* ranged from 92.5% to 98.9%, while phosphorus removal efficiencies ranged from 64.5% to 88.6% [81]. These results highlight the species’ capacity for nutrient recovery and its potential use in sustainable wastewater management strategies [81]. Furthermore, the proliferation of *Ulva* species in nutrient-rich coastal waters can be harnessed to mitigate eutrophication through nutrient bioextraction. By cultivating *Ulva* in nutrient-enriched waters, excess nitrogen and phosphorus can be absorbed and removed from circulation. This nutrient uptake may help to suppress the occurrence of harmful algal proliferations caused primarily by cyanobacteria and dinoflagellates, both of which respond strongly to eutrophication [82]. Reducing nutrient availability through *Ulva* cultivation can therefore mitigate the risk of cyanobacterial blooms (e.g., Microcystis *aeruginosa*) and dinoflagellate red tides (e.g., Alexandrium *tamarense*), thereby contributing to improved water quality [82,83]. For example, *U. lactuca* in the Marchica lagoon, Morocco, has shown the potential to bioaccumulate heavy metals and mitigate eutrophication [84]. Beyond nutrient removal, *Ulva* species contribute to carbon sequestration efforts. A study assessing the carbon sequestration potential of *Ulva* biomass estimated that it could sequester approximately 3.85 million tons of CO_2_ equivalents, with about 1.93 million tons potentially stabilized through biochar conversion [85]. This indicates the viability of integrating *Ulva* cultivation and biochar production into carbon management strategies [85].

Although *Ulva* species have considerable nutritional and economic potential, they also accumulate pollutants that limit safe utilization. It showed that *Ulva* acts as a hyperaccumulator of heavy metals such as Cd, Pb, Cu, Zn, and Hg [86]. Samples collected from industrially impacted coasts of Asia contained Cd and Pb concentrations that exceeded international food safety thresholds, which makes direct human or animal consumption unsafe [87]. The degree of metal accumulation depends on environmental factors such as salinity, nutrient status, and proximity to anthropogenic discharge. In estuarine and nearshore ecosystems subject to wastewater inflows or aquaculture effluents, *Ulva* thalli often contain several-fold higher metal concentrations compared with background seawater [86]. Such enrichment poses ecological risks because grazers and herbivorous fish ingest contaminated thalli. This process can trigger biomagnification, with heavy metal concentrations rising by about 10% at each trophic level. Beyond heavy metals, *Ulva* also retains microplastics and organic pollutants. These contaminants compromise the safety of food and feed applications and may intensify oxidative stress in consumers. Fish fed with *Ulva* collected from polluted waters exhibited elevated tissue concentrations of metals compared with control groups, which raises serious concerns about aquaculture use without strict monitoring [86]. Safe utilization of *Ulva* requires material collected from controlled environments such as integrated multi-trophic aquaculture systems, where water quality and contaminant inputs remain under regulation. Future work should develop standardized monitoring protocols for metal and pollutant concentrations in *Ulva* and assess their transfer potential within food webs.

## 4. Molecular Approaches in *Ulva* Taxonomy and Biodiversity Assessment

Traditional taxonomy of the genus *Ulva* has predominantly relied on morphological characteristics, including thallus architecture, cell arrangement, and reproductive structures. Species, such as *U. lactuca* are characterized by their broad, flat, and sheet-like thalli [12,68], whereas *U. intestinalis* exhibits tubular, hollow structures [7]. These morphological traits have historically served as primary identifiers in species classification. However, the reliance on morphological features presents significant challenges due to the high degree of phenotypic plasticity exhibited by *Ulva* species. Environmental factors can induce substantial morphological variations within the same species [30,35,36,37]. These variations often lead to misidentification. For instance, *U. compressa* and *U. intestinalis* can exhibit overlapping morphological features under varying environmental conditions [7]. This overlap complicates accurate species delineation. The limitations of morphology-based taxonomy are further highlighted by the presence of cryptic species within the genus. These are genetically distinct species that are morphologically indistinguishable. Studies have revealed that specimens identified morphologically as *U. lactuca* encompass multiple genetically distinct lineages. This finding indicates the presence of cryptic diversity within the species [8,49,68,71]. Such findings underscore the inadequacy of traditional taxonomic methods in capturing the true biodiversity within the genus. To address these challenges, there has been a shift towards integrating molecular techniques, such as DNA barcoding and phylogenetic analyses, into taxonomic studies of *Ulva*. These methods provide more reliable means of species identification by analyzing genetic markers [88,89]. This approach overcomes the limitations posed by morphological plasticity [90]. The adoption of molecular approaches has led to the reclassification of several *Ulva* species. This has also led to the discovery of previously unrecognized taxa (Table 2). These developments have improved our understanding of the genus’s diversity and evolutionary relationships.

For instance, analyses of the *U. lactuca* complex using chloroplast ribulose-1,5-bisphosphate carboxylase/oxygenase large subunit (*rbcL*) and nuclear Internal Transcribed Spacer (ITS) markers have revealed multiple genetically distinct lineages [6,93]. These lineages were found within specimens previously identified as *U. lactuca* [8]. This finding indicates the presence of cryptic species. Similarly, research on *U. compressa* has demonstrated significant genetic divergence among morphotypes collected from different geographic regions, despite their morphological similarities [7]. The recognition of cryptic diversity in *Ulva* has profound implications for ecological studies and resource management. Misidentification of species can lead to inaccurate assessments of biodiversity, misinterpretation of ecological roles, and ineffective management strategies. For example, different cryptic species may exhibit varying responses to environmental stressors [7]. These differences can affect their proliferation and ecological impact. Accurate species identification is therefore crucial for monitoring algal blooms, managing aquaculture practices, and conserving marine ecosystems.

By comparing sequence data across populations, we can uncover previously unrecognized lineages, assess gene flow, and determine whether morphological similarity results from shared ancestry or convergent adaptation to similar environments. In *U. lactuca*, for example, barcoding studies have revealed that what was once considered a single, globally distributed species actually comprises several genetically distinct taxa that differ significantly in their ecological niches and geographic ranges [9,68]. Similarly, *U. compressa* and *U. intestinalis*, long recognized as separate species based on thallus form, have shown overlapping morphological traits but distinct genetic profiles when analyzed with ITS and *rbcL* sequences [7].

Additionally, molecular taxonomy has also led to the formal reclassification of certain species and the description of new ones. For instance, *U. ohnoi* was originally misidentified as part of the *U. lactuca* complex based on its flattened blade-like appearance, but molecular phylogenetic analysis placed it as a distinct lineage with separate ecological characteristics and regional distribution [6,62]. Furthermore, molecular investigations in East Asian waters have uncovered novel species, such as *U. paschima* Bast, identified using elongation factor Tu (*tufA*) and ITS barcoding in specimens collected from the Indian west coast [24]. These examples illustrate how molecular data have significantly expanded our understanding of *Ulva* biodiversity and corrected long-standing taxonomic errors rooted in morphological interpretation alone. Recent advances in molecular genetics have significantly improved our understanding of *Ulva* biodiversity and taxonomy. Commonly used DNA markers such as ITS, *rbcL*, and *tufA* have been widely applied for phylogenetic analyses, with *tufA* emerging as the most consistent marker across species delimitation methods [92]. Algorithmic approaches such as the Generalized Mixed Yule Coalescent and Poisson Tree Processes have further refined species boundaries, though conflicts remain among markers and with traditional morphological classifications [92]. Genomic resources, including complete chloroplast and mitochondrial genomes, are increasingly available and provide multilocus data for resolving taxonomically challenging clades (Table 3). These molecular approaches complement morphological and reproductive studies and are essential for addressing cryptic diversity and achieving a stable taxonomic framework for *Ulva*.

Population genomics, enabled by high-throughput sequencing technologies, allows for the examination of genome-wide variation across natural populations. This approach provides insights into local adaptation, gene flow, and population structure, which are critical for understanding the evolutionary dynamics of *Ulva* in response to environmental pressures [9,93]. Studies analyzing chloroplast genome variation have revealed substantial differences in gene content, structure, and sequence divergence among closely related *Ulva* taxa [25]. These differences not only support the delineation of cryptic species but also inform phylogeographic patterns and the evolutionary history of the genus [6,24]. Moreover, pan-genome approaches have been proposed to capture the full complement of core and accessory genes within *Ulva* species. For example, the genome sequencing and annotation of *U. compressa* provides substantial insights that enhance the molecular perspective on *Ulva* species [94]. With a genome size of 80.8 Mb and 19,207 predicted protein-coding genes, *U. compressa* possesses a higher gene count than *U. mutabilis*, which contains 12,924 genes (Table 2). This suggests a greater gene density and complexity than previously expected for this genus. The presence of multiple antioxidant enzyme genes, particularly eleven ascorbate peroxidase genes and the absence of catalase, highlights a unique oxidative stress response mechanism. This supports the physiological evidence of *Ulva*’s high tolerance to heavy metals. In addition, the genome reveals a diverse array of signaling components, including 76 protein kinases such as mitogen-activated protein kinases, calcium-dependent protein kinases, and calcium/calmodulin protein kinases. The absence of ethylene and abscisic acid biosynthetic enzymes, combined with the presence of their precursor synthesis genes and hormone-responsive transcription factors, indicates the possibility that *Ulva* uses 1-aminocyclopropane carboxylic acid and abscisic acid-aldehyde as signaling molecules [94].

Recent advances in molecular taxonomy, particularly high-throughput sequencing and genome-wide analysis, have not only enhanced species resolution but also opened new avenues for applied *Ulva* research [5,12]. Population genomic approaches can identify adaptive genetic variants linked to salinity, nutrient availability, or temperature tolerance [28,38,47], which are critical for selecting strains suited to specific aquaculture environments. This supports the development of targeted breeding programs aimed at optimizing growth rates, biomass yield, and biochemical composition [99]. In addition, genomic surveillance of bloom-forming species enables early detection and tracking of genetic lineages associated with harmful “green tides” [100]. By monitoring genetic shifts within populations, researchers and policymakers can predict bloom events more accurately and implement proactive coastal management strategies. For example, distinguishing cryptic bloom-forming taxa such as *U. prolifera* from non-prolific relatives allows for more refined modeling of bloom dynamics under varying environmental conditions [11,30]. By bridging molecular resolution with ecological function and commercial potential, these tools enhance our ability to cultivate, manage, and conserve *Ulva* resources in line with blue bioeconomy goals.

## 5. Future Perspectives

Future research on *Ulva* taxonomy should focus on approaches that combine morphology, molecular markers, and ecological data. Morphological traits remain valuable for field identification but often fail to distinguish cryptic species. Molecular methods such as DNA barcoding, phylogenetics, and genomics provide greater accuracy but can give incomplete results if used in isolation. A framework that integrates these tools will improve biodiversity assessment, species monitoring, and ecological analysis. In aquaculture, conservation, and coastal management, accurate taxonomy will strengthen decisions and support resource management. Standardized protocols that include both morphology and genetic markers would reduce taxonomic uncertainty and support long-term applications.

Recent advances in genome sequencing provide opportunities to identify adaptive traits, resolve cryptic diversity, and uncover molecular mechanisms underlying environmental tolerance. Future studies should develop genomic resources across a wider range of *Ulva* taxa and apply pan-genome and population genomic approaches to understand intraspecific variation, local adaptation, and evolutionary trajectories. Although ulvan has been widely studied, the full diversity of *Ulva* metabolites and their bioactivities remains underexplored. High-resolution metabolomics and functional assays should be used to discover novel compounds of pharmaceutical and nutraceutical interest. Standardized extraction and structural characterization protocols will be important for translating laboratory findings into industrial applications. Additionally, optimized cultivation strategies, including integrated multi-trophic aquaculture, offshore farming, and photobioreactor systems, need further development for scalable biomass production. Future research should explore combined biorefinery approaches that simultaneously generate food, feed, energy, and biomaterials from the same *Ulva* biomass, enhancing sustainability and economic value. Meanwhile, understanding how *Ulva* responds to changing environmental factors, such as temperature shifts, salinity fluctuations, eutrophication, and ocean acidification, is essential. Multi-stressor experiments that combine physiological assays with transcriptomics will help predict how *Ulva* populations and green tides may shift under climate change scenarios. Given its capacity to accumulate nutrients and heavy metals, *Ulva* can serve as a sentinel organism for environmental change. Future work should integrate *Ulva* into biomonitoring frameworks for coastal ecosystems, using molecular and biochemical markers to improve sensitivity and accuracy. Finally, *Ulva*’s dual role, as both an ecological engineer and a contributor to nuisance blooms, necessitates a balanced approach. Collaborative studies between ecologists, economists, and policymakers are needed to design sustainable harvesting strategies, valorize bloom biomass, and align *Ulva*-based industries with circular bioeconomy and climate adaptation policies.

## 6. Conclusions

*Ulva* species play an important role in marine, brackish, and freshwater ecosystems due to their ecological adaptability, rapid growth, and biochemical diversity. They provide ecosystem services such as nutrient cycling, habitat provision, and bioindication, and they offer opportunities for applications in food, feed, bioremediation, biofuels, pharmaceuticals, and biomaterials. However, high phenotypic plasticity complicates taxonomy and demands the integration of molecular approaches. Nutrient removal shows promise for wastewater treatment, yet efficiency depends strongly on environmental conditions, and large-scale field applications are still rare. The use of *Ulva* in food and feed chains raises safety concerns because thalli can accumulate heavy metals, microplastics, and organic pollutants that pose risks of biomagnification. Barriers to biofuel and biomaterial production include low lipid content, high processing costs, and the absence of standardized extraction methods. Future research must close these gaps by linking molecular genetics with physiology and applied studies to refine taxonomy, optimize cultivation systems, and discover new bioactive compounds. Freshwater representatives also deserve more attention, as they extend the ecological amplitude of the genus and remain underexplored. Establishing international safety standards and monitoring protocols will be essential for the responsible use of *Ulva*. Taken together, the genus should be regarded as both a promising bioresource and a potential ecological risk factor, and only a balanced perspective that considers both benefits and limitations will allow *Ulva* to contribute effectively to biodiversity studies, environmental management, and bioeconomic development.

## Figures and Tables

**Table 1:** Summary of the main applications of *Ulva* biomass in food, feed, bioremediation, biofuel production, pharmaceuticals, and material science.

#	Application	Advantages	Disadvantages
1	Human food	Rich source of proteins, polysaccharides (ulvan), vitamins, and minerals; traditional use in coastal diets; bioactive compounds with antioxidant properties	Risk of heavy metal, microplastic, and pollutant accumulation; variable nutritional composition depending on environment; sensory acceptability issues
2	Animal feed	Enhances growth performance, antioxidant status, and immune response; reduces oxidative stress; potential to replace fishmeal in aquaculture	Contaminated thalli may transfer heavy metals to higher trophic levels; digestibility and palatability challenges at high inclusion rates
3	Bioremediation	Effective uptake of nitrogen and phosphorus; accumulation of heavy metals; potential for integration in aquaculture systems	Effective uptake of nitrogen and phosphorus; accumulation of heavy metals; potential for integration in aquaculture systems
4	Biofuel and bioenergy	High carbohydrate content suitable for bioethanol and biogas production; renewable and sustainable resource	Low lipid content limits biodiesel potential; preprocessing and conversion technologies are costly; economic viability not yet proven
5	Pharmaceuticals and nutraceuticals	Ulvan and other metabolites show antiviral, antibacterial, anticoagulant, and immunomodulatory activities; potential for functional food ingredients	Lack of standardized extraction and purification methods; variability in bioactivity between species and locations; limited clinical validation

**Table 2:** Comparison of traditional and molecular taxonomic approaches used in the classification of *Ulva* species. Data were obtained and adapted from recent studies, with corresponding bibliographic references provided [7,24,26,58,91,92].

#	Criteria	Traditional Taxonomy	Molecular Taxonomy
1	Diagnostic basis	External morphology (e.g., thallus shape, size, branching)	Genetic sequences (*rbcL*, ITS, *tufA*)
2	Accessibility	High, requires minimal equipment	Limited, requires laboratory infrastructure
3	Cost	Low	Moderate to high
4	Speed	Rapid in field settings	Slower due to DNA extraction, PCR, and sequencing
5	Resolution in cryptic species	Poor, often fails to separate genetically distinct species	High, reveals cryptic lineages within morphologically similar taxa
6	Sensitivity to environment	High, morphology varies with conditions	Low, genetic identity remains stable
7	Suitability for biodiversity surveys	Useful for rapid assessments	Necessary for precise species inventories
8	Examples of challenges	*U.* lactuca vs. U. *fasciata*; *U.* compressa vs. U. *intestinalis*	Resolution of *U. ohnoi*, *U. paschima*, and *U. gigantea* complexes

**Table 3:** Summary of publicly available genome assemblies for selected *Ulva* species. Data was retrieved from the NCBI GenBank/Genome database (BioProject accession numbers are provided), with bibliographic references included where available.

#	Genome Assembly	Accession	Species	Genome Size (Mb)	References
1	ASM2307855v1	PRJNA705045	*U. prolifera* isolate: Qingdaoensis	88.9	-
2	ASM2450001v1	PRJNA824527	*U. compressa* isolate: HO_PTR_2022v1	80.8	[94]
3	Ulvmu_WT_fa	PRJEB25750	*U. mutabilis* strain wild-type	98.5	[95]
4	ASM3236096v1	PRJNA894635	*U. armoricana* isolate: IRGN 2022FHL053	127.7	[96]
5	U_prolifera_HU1.0	PRJDB13471	*U. prolifera* isolate: Hashirijima_01	103.8	[97]
6	ASM413825v1	PRJNA484545	*U. prolifera* Isolate: YS-2018	87.9	[98]

## Data Availability

Data sharing is not applicable to this article as no new data were created or analyzed in this study.
